# Flexible Toolbox of High-Precision Microfluidic Modules for Versatile Droplet-Based Applications

**DOI:** 10.3390/mi15020250

**Published:** 2024-02-07

**Authors:** Mario Saupe, Stefan Wiedemeier, Gunter Gastrock, Robert Römer, Karen Lemke

**Affiliations:** 1 Institute for Bioprocessing and Analytical Measurement Techniques e.V., 37308 Heilbad Heiligenstadt, Germany; stefan.wiedemeier@iba-heiligenstadt.de (S.W.); gunter.gastrock@iba-heiligenstadt.de (G.G.); robert.roemer@iba-heiligenstadt.de (R.R.); karen.lemke@iba-heiligenstadt.de (K.L.); 2 Department of Physical Chemistry and Microreaction Technologies, Technical University of Ilmenau, 98693 Ilmenau, Germany

**Keywords:** droplet-based microfluidics, high throughput, droplet generation, mixing, merging, active fluid injection, droplet content analysis, cell-based assay

## Abstract

Although the enormous potential of droplet-based microfluidics has been successfully demonstrated in the past two decades for medical, pharmaceutical, and academic applications, its inherent potential has not been fully exploited until now. Nevertheless, the cultivation of biological cells and 3D cell structures like spheroids and organoids, located in serially arranged droplets in micro-channels, has a range of benefits compared to established cultivation techniques based on, e.g., microplates and microchips. To exploit the enormous potential of the droplet-based cell cultivation technique, a number of basic functions have to be fulfilled. In this paper, we describe microfluidic modules to realize the following basic functions with high precision: (i) droplet generation, (ii) mixing of cell suspensions and cell culture media in the droplets, (iii) droplet content detection, and (iv) active fluid injection into serially arranged droplets. The robustness of the functionality of the Two-Fluid Probe is further investigated regarding its droplet generation using different flow rates. Advantages and disadvantages in comparison to chip-based solutions are discussed. New chip-based modules like the gradient, the piezo valve-based conditioning, the analysis, and the microscopy module are characterized in detail and their high-precision functionalities are demonstrated. These microfluidic modules are micro-machined, and as the surfaces of their micro-channels are plasma-treated, we are able to perform cell cultivation experiments using any kind of cell culture media, but without needing to use surfactants. This is even more considerable when droplets are used to investigate cell cultures like stem cells or cancer cells as cell suspensions, as 3D cell structures, or as tissue fragments over days or even weeks for versatile applications.

## 1. Introduction

The term “droplet-based microfluidics” refers to techniques that involve handling discrete fluid samples with volumes ranging from picoliters up to a few microliters. Opposite to techniques like “hanging drop” or “liquid overlay” [1,2], where the fluid samples are separated in wells or by special domains, in droplet-based microfluidics, the fluid samples are situated in a micro-channel and separated by a second fluid, which is immiscible with the fluid samples. As the fluid samples (also named “droplets” or “disperse phase”) normally have watery properties and consequently a high polarity, the second fluid has to have non-polar properties to avoid the merging of droplets. Consequently, for this second fluid, often named the “continuous phase”, oils are commonly used. Other workgroups use surfactants to decrease the surface tension of the fluid as well as the droplet volumes [3]. Droplet-based microfluidics offer high potential for a broad range of applications in biotechnology [4], life sciences [5,6], as well as in chemistry [7,8,9,10] or combinations of that with the synthesis of superparamagnetic iron oxide nanoparticles (SPIONs) for diagnostic and therapeutic applications [10]. For example, for applications in the field of multi-parametric analysis, it has advantages in contrast to microplate technologies [11]. Compared to the latter, droplet-based microfluidics work with significantly smaller sample volumes, which is important, e.g., in the case of experiments using patient samples. As a consequence, the volumes of reagents could also be decreased, resulting in an additional reduction in costs [12]. Furthermore, the automation of microplate technologies requires the application of expensive and laboratory space-consuming pipetting robots. There is an enormous potential for droplet-based microfluidics regarding the reduction in costs and the increase in further applications [13]. Experiments based on droplet-based microfluidics are usually performed in micro-channels and consequently in closed systems. For biological or biomedical applications, e.g., with cells, this feature is advantageous because it minimizes the danger of contamination for both the cell culture and the experimenter. Additionally, in comparison with microplates, the evaporation of droplets is negligible, which increases the range of applications as well.

Microfluidic chips and modules are mainly manufactured from glass, silicon, and polymers. The manufacture of polymers has some advantages compared to etching procedures [14,15] and can be produced by the LIGA process, PDMS technology, injection molding, rapid prototyping, and much more [16]. To avoid droplets sticking at the micro-channel, its surface has to have hydrophobic properties, corresponding with low surface energy. This could be guaranteed by using hydrophobic polymers like polytetrafluoroethylene (PTFE) or fluoroethylenepropylene (FEP) or by sealing the surface with a hydrophobic layer by silanization, plasma treatment [16,17], sol–gel coating [18] or the deposition of soot [19] or nanoparticles [20]. As many fluidic operations such as droplet generation, droplet splitting, droplet sorting, as well as mixing and merging have been developed and established in recent years, a lot of biological, chemical, and medical processes can be performed [10,21,22,23,24,25,26,27,28].

Our microfluidic modules are easy and cost-effective to manufacture by injection molding and milling. The injection-molded chips have a micro-structure that is transferred from their mold to the modules, which are plasma-treated as well. This leads to better reproducibility and long-lasting use compared to milled modules, which are only plasma-treated. Both can be used as disposables, on the one hand, and hydrophobized, which ensures that they are reusable, on the other hand. The hydrophobic surface is even more important when the experiments are to be performed without the use of surfactants due to biocompatibility, as is the case using the “*pipe-based bioreactors (pbb)”* platform, to avoid unintended droplet merging and realize the long-term cultivation of cells and 3D cell structures as a special application among others [17]. Because the storage module (SM) consists of PTFE tubes, which are semipermeable for gases, there is no limitation in cell cultivation in contrast to the various 3D-printed systems reviewed by Lai et al. [29].

**Figure 1:**
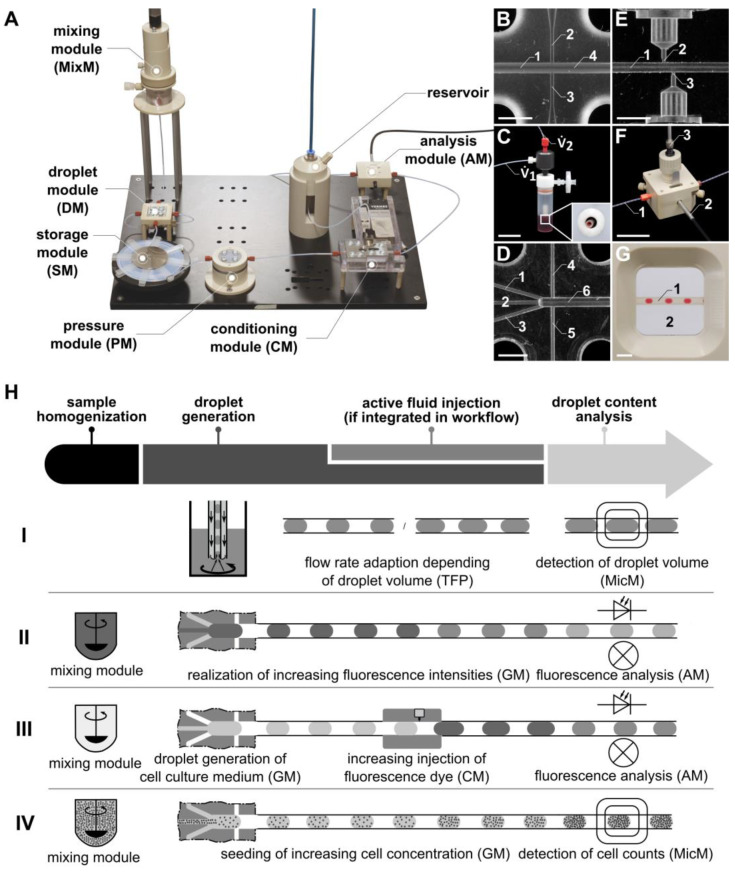
(**A**) Photo of the “*pbb*” system, with the newly developed microfluidic modules: gradient module (GM), conditioning module (CM), and analysis module (AM). In place of or in addition to the AM, a microscopy module (MicM) (**G**), connected to a microscope, can be integrated. (**B**) Channel structure of the droplet module (DM) fluid micro-system (FMS). 1: inlet for the disperse phase; 2, 3: inlets for the continuous phase; 4: outlet. (**C**) Photo of the Two-Fluid Probe (TFP). (**D**) Channel structure of the GM FMS. 1, 2, 3: inlets for the disperse phase; 4, 5: inlets for the continuous phase; 6: outlet. (**E**) Channel structure of the CM FMS. 1: main channel for droplet transport; 2, 3: side channels for piezo valve connection and fluid injection, respectively. (**F**) Photo of the AM FMS. 1: FEP tube for droplet transport; 2: optical fiber for the transport of the excitation light; 3: optical fiber for the transport of the emitted fluorescence light. (**G**) Detail of the MicM FMS. 1: FEP tube with droplets; 2: chamber filled with immersion fluid. (**H**) Schematic illustration of the workflow using the modular microfluidic platform generating droplets with the TFP (see **H**, row I), passive mixing of fluorescence dye (see **H**, row II), and cell suspension (see **H**, row IV) with the GM and active fluid injection with the CM (see **H**, row III). Scale bars: (**B**,**D**,**E**,**G**) = 3 mm: (**C**,**F**) = 30 mm.

In this manner, it is possible to use microchips for droplet generation from cell culture medium, e.g., Dulbecco’s Modified Eagle Medium (DMEM), separated by an oil like perfluorodecaline (PFD) [26]. In that case, the droplets could serve as “bioreactors” separated by the biologically inert PFD. We use those bioreactors for biological and biomedical applications, e.g., for the cultivation of cells, bacteria, and viruses, and incubate them in pipes, e.g., in PTFE tubes with inner diameters ranging from 250 µm up to 3.2 mm. This also leads to droplet volumes in a suitable range of 60 nL to 20 µL, which is higher as compared to a system presented by Chen et al. [30]. This is the origin of “*pipe-based bioreactors* (*pbb)”* (Figure 1), a brand registered by iba e.V. for its technique of dealing with droplet-based microfluidics [31,32]. From discussions with cooperating medical scientists, it is an advantage to use no surfactants in biological and medical approaches [33]. The difficulty is in avoiding droplet contact in order to prevent unintended droplet merging.

Nevertheless, we focused on the development of a droplet-based microfluidic platform, working without such surfactants, and developed methods to overcome this problem and guarantee stable droplet sequences [17,27]. In contrast to that, lower droplet volumes are pre-destined for single-cell analysis as well as faster droplet generation by the use of surfactants [34]. In both cases, the flow rate ratio affects the droplet size [35]. In contrast to flow focusing and T-junction solutions, the presented Two-Fluid Probe (TFP) is pre-destined to generate droplets from a homogenous solution, including chemicals or cell suspensions, without the need of an additional microfluidic mixer. Cell concentrations can be easily varied by changing the connected vessel, in which the cell suspension is stored aseptically. Furthermore, the TFP allows for easy adaption of the droplet volume by changing to PTFE tubes with different diameters. As a result, it is more adaptable and easier to produce compared to chip-based microfluidic modules. Here, the influence of varying flow rates resulting in different droplet volumes and distances between them is described. The robustness of the droplet generation using the TFP is tested here for the first time. With the development of new modules, the accuracy of mixing of different fluids (for example, cell suspension with medium or drugs) before droplet generation, on the one hand, and merging of existing droplet sequences with fluids (for example, medium, drugs, and dyes), on the other hand, are examined.

In this study, we present a modular droplet-based platform, the “*pbb*” system, that consists of different microfluidic modules connected through PTFE tubes. The combination of different microfluidic modules is shown to develop different process steps, realizing cell-based approaches in long-term experiments. Adjusting the length of the PTFE tubes enabled the handling of a flexible number of samples, while in systems like the LEGO^®^-like system, only a fixed number of samples can be managed [36]. The highly reproducible cultivation of embryoid bodies, food analysis, nanoparticle synthesis, and droplet generation from blood already emphasized the importance of the “*pbb*” system [10,26,27,28]. The flexibility in combining the different modules and their high-precision functionality will enable the realization of diverse biological, chemical, and medical applications in the future.

## 2. Materials and Methods

The potential of the “*pbb*” platform is demonstrated by highlighting specific applications pertaining to the generation, mixing, merging, and investigation of droplets. These applications are particularly relevant for the cultivation of cell suspensions in droplets and utilize functional models within the “*pbb*” platform (see Figure 1A). While certain modules have been previously described in [27,28], this paper introduces novel modules for the first time, contributing to the expanding capabilities of the “*pbb*” platform.

### 2.1. Solutions and Chemicals

For the characterization of the TFP, DMEM cell culture medium (D 5523, Sigma-Aldrich Chemie GmbH, Taufkirchen, Germany) supplemented with 5.5 g L^−1^ D-glucose (G7021, Sigma-Aldrich Chemie GmbH, Taufkirchen, Germany), 2 mM L-glutamine (G8540, Sigma-Aldrich Chemie GmbH, Taufkirchen, Germany), 100 U penicillin/100 µg mL^−1^ streptomycin (15140-122, Life Technologies, Darmstadt, Germany) and 10% [*v*/*v*] fetal calf serum (FCS) (S1810-500, Biowest, Nuaille, France) was used as the dispersed phase.

For all other experiments, MEM cell culture medium (21090-055, Life Technologies GmbH, Darmstadt, Germany) was used. The MEM was supplemented with 10% [*v*/*v*] FCS (S1810-500, BioWest, Nuaille, France), 1 mM sodium pyruvate (11360-039, Life Technologies GmbH, Darmstadt, Germany), 0.1 mM non-essential amino acids (11140-035, Life Technologies GmbH, Darmstadt, Germany), 0.75 g sodium bicarbonate (S5761, Sigma-Aldrich Chemie GmbH, Taufkirchen, Germany), 100 U penicillin/100 µg mL^−1^ streptomycin (15140-122, Life Technologies, Darmstadt, Germany), and 0.1% [*v*/*v*] phenol red (P5530, Sigma-Aldrich Chemie GmbH, Taufkirchen, Germany).

The resorufin sodium salt powder (424455-1G, Sigma-Aldrich Chemie GmbH, Taufkirchen, Germany) was dissolved in MEM cell culture medium at a final concentration of 0.1 mg mL^−1^. As the continuous phase, PFD (A18288, ALFA AESAR, Karlsruhe, Germany) was used in all experiments.

### 2.2. Glioblastoma Cell Line U87MG ATCC

For cell-based experiments, the glioblastoma cell line U87MG ATCC (ATCC-HTB-14, LGC Standards GmbH, Wesel, Germany) was used. Tumors of the central nervous system were classified from the World Health Organization (WHO) in the year 2000 [37], with glioblastoma designated as WHO grade IV—the most aggressive form of brain tumors [38]. The average life expectancy for individuals with this tumor type is about 14.6 months [39]. To advance strategies against this disease, we investigated the U87MG ATCC glioblastoma cell line. The cells were cultured in MEM medium (see previous chapter) without additional phenol red. Cells were subcultured twice a week at a 1:10 ratio, maintaining a maximum confluence of 80%. Only cell suspensions with a viability exceeding 95% were used for the experimental analyses. 

### 2.3. Fabrication of the Fluid Micro-System-Based Modules

All fluid micro-system (FMS)-based modules consist of two halves, manufactured from polymer material (polycarbonate, PC) by precision mechanical procedures. Each half of the module possesses identical semi-circular channel structures, to form a microfluidic structure with round channels and hydrophobic surface properties. FMS-based modules were integrated into a frame to facilitate tube connections. Depending on the fluid connections, the system can be used for different operations. Following octafluorcyclobutane plasma coating (30 min, 80 W, 15 sccm) for micro-channel hydrophobization, as previously described [17], the two module halves are securely fastened together.

### 2.4. Microfluidic Modules

#### 2.4.1. Droplet Generation Modules

**Figure 2:**
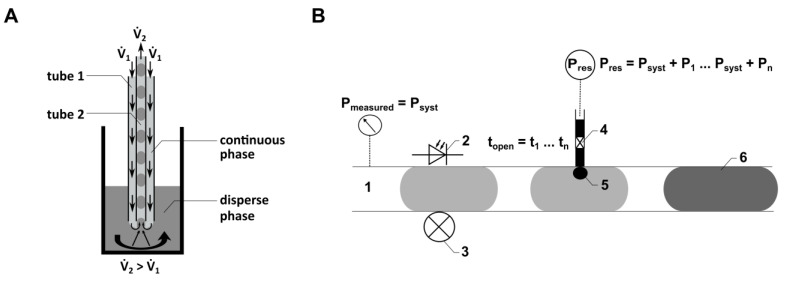
(**A**) Schematic illustration of the principle of the TFP. $\dot{V}$1: positive flow rate for continuous phase. $\dot{V}$2: negative flow rate for displacing disperse phase from the reaction vessel. (**B**) Schematic illustration of the droplet injection process using the CM. 1: location of the pressure sensor in the micro-channel; 2, 3: location of the droplet detection in the CM FMS by using optical fibers; 4: piezo valve connected to a reservoir with a defined higher pressure compared to the pressure in the micro-channel; 5: injection area; 6: droplet, including injected volume.

We used two kinds of microfluidic modules for droplet generation: (i) FMS-based modules and (ii) probe-based modules. The FMS-based modules (Figure 1B) were developed and investigated by Wiedemeier et al. with detailed descriptions in [27]. These modules are referred to as droplet modules (DMs). The probe-based modules implement the principle of droplet generation using a probe composed of two nested tubes, as introduced by Schemberg et al. as the Two-Fluid Probe (TFP) (see Figure 1C, [28]). The TFP is based on the design of a double lumen catheter, well-known from medical applications [40], which will be described in more detail in the following sections. In contrast to the most common strategies using T-junctions and flow-focusing geometries to generate droplets, the TFP is particularly suited to generate droplets from vessels (e.g., bioreactors or wells of microplates) directly into the inner tube of the TFP (Figure 2A). As the droplets (samples) are enveloped within a continuous phase like PFD, there is no dead volume, allowing for a significant reduction in sample volume. 

Here, droplet generation was performed continuously with both flow rates ($\dot{V}$1 and $\dot{V}$2) activated. The local flow field at the interface between the dispersed phase (cell culture medium) and the continuous phase (PFD) influences both the droplet volume and the distance between the droplets. The droplet generation is determined by the relationship between viscous shear stresses, which deform the fluid interface (caused by $\dot{V}$1), and the force resulting from capillary pressure superposed by $\dot{V}$2. Quantitative predictions of droplet formation parameters, like the droplet volume or the distance between the droplets, can be performed analytically or numerically [41]. As the droplet generation probe (Figure 2A) can be manufactured from thermally stable materials, sterilization methods such as autoclaving can be applied. This characteristic is crucial for using the probe in droplet-based sampling from bioreactors. Flow rates ranging from 33 µL min^−1^ to 80 µL min^−1^ were employed for $\dot{V}$1, while a consistent flow rate of −89.4 µL min^−1^ was used for $\dot{V}$2.

#### 2.4.2. Gradient Module (GM)

Both the FMS-based module [27] and the probe-based module [28] were developed to generate droplets from single-phase fluids, like cell suspensions. However, for the investigation of cell cultures in droplets, it is necessary to generate droplets with varying cell concentrations. To address this, the GM was developed. In contrast to the FMS-based flow-focusing droplet generation module [27], which features three inlets for the single-phase fluids and one outlet for the droplet sequence, the GM features five inlets and one outlet (Figure 1D). Here, it is possible to mix different chemicals or cell suspensions by pumping them through three channels and subsequently generating droplets by flow focusing using one module. Drug screening investigations using droplet-based microfluidics require both the generation of droplets with different but defined cell counts and the handling of different but defined concentrations of drugs. Both of these requirements were realized using the GM, with a flow rate of 500 µL min^−1^ for the continuous phase and a flow rate of 100 µL min^−1^ for the dispersed phase.

#### 2.4.3. Conditioning Module (CM)

To inject fluids actively into droplets, a novel module called CM (Figure 1A) was developed. It allows the injection of dyes, drugs, and other chemicals into a droplet sequence. The CM consists of four components connected by PTFE tubes (Jasco GmbH, Gotha, Germany) with an inner diameter of 1000 µm. These four components include a pressure module (PM), an injection FMS, the piezo valve, and a reservoir (see Figure 1A) to store the fluid to be injected into the droplets.

A PM is required to monitor the pressure within the micro-channels of the droplet-based microfluidic system (Figure 1A). To realize fluid flow without disturbing the droplet sequence, an FMS was developed. It consists of a main channel (1000 µm diameter) and a side channel (500 µm diameter). The side channel connects the main channel to the pressure sensor. For pressure measurement, a PEEK connector was used, which has a cavity filled with the continuous phase to transfer the pressure inside the main channel, where the droplets are guided through, to the pressure sensor.

The injection FMS (Figure 1E) consists of a main channel (1000 µm diameter) where the droplets are guided through and two side channels (300 µm diameter), each connecting the nozzle of a piezo valve (VERMES Microdispensing GmbH, Holzkirchen, Germany) directly to the main channel. The piezo valves are positioned within a PEEK-made connection frame, realizing the connection to the side channels of the FMS. An optical light barrier integrated into the FMS, consisting of an optical fiber, a photodiode and a laser diode, is used to detect the position of droplets traversing the main channel of the FMS. Knowing the fluid flow (conventional flow rate of 300 µL min^−1^) and the distance between the optical light barrier and the side channel, the point in time is calculated when the droplet position aligns with the side channel, triggering the piezo valve for fluid injection (see schematic illustration in Figure 2B).

The reservoir contains the fluid to be injected by the piezo valve into the droplets. It is connected to a compressed air supply, which guarantees a difference between the pressure inside the main channel of the FMS and the pressure inside the reservoir. This pressure difference as well as the opening time of the piezo valve can be set by the control unit of the droplet-based system and the control unit of the piezo valve (VERMES Microdispensing GmbH, Holzkirchen, Germany), respectively.

#### 2.4.4. Analysis Module (AM)

**Figure 3:**
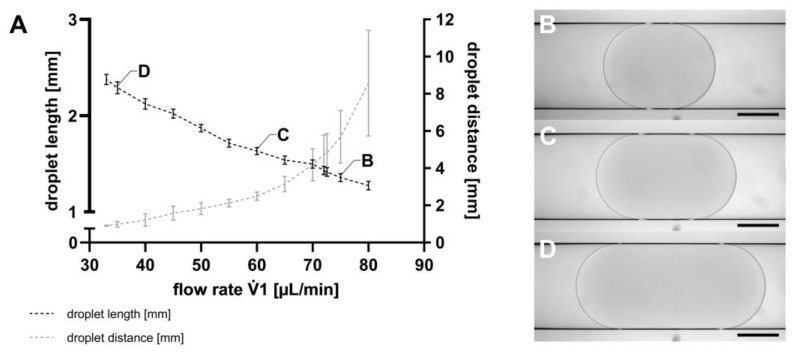
(**A**) Droplet length (black) and distance between the droplets (grey) shown as mean with SD, n = 100. The flow rate $\dot{V}$1 of the TFP was changed, whereas the flow rate $\dot{V}$2 was constant at −89.4 µL min^−1^ for all flow rates $\dot{V}$1. Droplets were visualized using the MicM connected to a BX60 microscope (Olympus Deutschland GmbH, Hamburg, Germany) generated with (**B**) $\dot{V}$1 = 75 µL min^−1^; droplet length (L) = 1344 µm, (**C**) $\dot{V}$1 = 60 µL min^−1^; L = 1673 µm, and (**D**) $\dot{V}$1 = 35 µL min^−1^; L = 2240 µm. Scale bar = 500 µm.

Here, we present an optical flow sensor for continuous droplet detection with interfaces to connect a spectrometer via optical fibers (Figure 1F). The resorufin excitation was realized using a laser diode emitting at a wavelength of 525 nm or a light source (ILP-2, Olympus Deutschland GmbH, Hamburg, Germany) combined with a filter (554 nm), respectively. The light was transferred via an optical fiber to the resorufin droplets (Figure 1F, 2), continuously guided through the AM by the syringe pump (Nemesys, Cetoni GmbH, Korbußen, Germany) (Figure 1F, 1). The fluorescence emitted from the resorufin suspension was transmitted to a spectrometer (STE-Silver-Nova, StellarNet Inc., Tampa, FL, USA) via an optical fiber (Figure 1F, 3). To avoid the influence of scattered light caused from the excitation light, an emission filter (590 nm) was installed. The optical fiber was oriented at a right angle to the optical fiber for the excitation light. For real-time analysis of the fluorescence light using the PC-based software “SpectraWiz” (version 5.3.3, StellarNet Inc., Tampa, FL, USA), the spectrometer was connected to a PC via USB. To improve the quality of the optical detection, the AM was equipped with an FEP-tube through which the droplets passed during the detection process, with a flow rate of 50 to 100 µL min^−1^. Additionally, the specific module design allowed filling the space between the FEP tube and the optical fibers with an immersion fluid, such as water, to reduce refraction caused by different refractive indices.

#### 2.4.5. Microscopy Module (MicM)

Similar to the AM, the MicM (Figure 1G) was equipped with an FEP tube to optimize microscopic imaging. The MicM can also be filled with an immersion fluid like water to minimize refraction due to different refractive indices. For this, two cover slips sealed with O-rings create a flat chamber, enabling compatibility with microscope objectives with a minimum working distance of 1.5 mm (focused onto the bottom of the inner diameter of the FEP tube). The droplets were guided through the FEP tube with a flow rate of 50 µL min^−1^.

### 2.5. Measurement of Droplet Lengths with the MicM

To measure injected volumes into the droplets, the MicM was used. For this, the MicM was filled with water (see Section 2.4, Microscopy Module) and droplets were detected using an optical detection system consisting of a camera (Basler AG, Ahrensburg, Germany) and an area light source (LUMIMax, iiM AG measurement and engineering, Suhl, Germany). 

The camera software (MedeaLab (version 1.0), Medea AV Multimedia and Software GmbH, Nürnberg, Germany) displays a region of interest (ROI) focused on the center of the FEP tube. During the passage of a droplet through the ROI, its length was recorded. As the shape of a droplet can be described as a cylinder with convex ends, the volumes of droplets, which were longer than the tube diameter (1 mm), could be determined using Equation (1). Equation (2) was used to calculate the volumes of the droplets smaller than the tube diameter.

V_(L > D) = ((π × D²)/4) × (L − D) + ((D^3^ π))/6 (1)

V_(L < D) = ((D^3^ π))/6 (2)

V—droplet volume;
D—droplet diameter;
L—droplet length.

### 2.6. Generation of Different Defined Droplet Volumes and Distances by Variation of $\dot{V}$1 Flow Rate in the TFP

To generate droplets of varying volumes and distances for specific applications, the TFP was used. In the experiments, the flow rate $\dot{V}$1 was changed from 33 µL min^−1^ to 80 µL min^−1^ at a constant flow rate for $\dot{V}$2 at −89.4 µL min^−1^ (Figure 2A). To determine the lengths (or volumes) of the droplets and the distances between them, dependent on $\dot{V}$1 and $\dot{V}$2, the droplet sequences were guided through the MicM (Figure 1G), optimized for microscopic droplet characterization (see Section 2.5). For enhanced optical imaging, the PTFE tube within the MicM was replaced with an FEP tube of the same dimensions. The droplets were measured using the MedeaLab software (version 1.0) from the Medea AV Multimedia and Software GmbH, Nürnberg, Germany.

### 2.7. Calibration of the AM Using a Fluorescence Gradient

To characterize the AM, the mixing module (MixM) was filled with a resorufin solution at 100 µg mL^−1^ (see Figure 1A) and droplets with varying end concentrations were generated. The transport of the resorufin solution was achieved by displacing it with Tetradecane (TD) (172456, Sigma-Aldrich Chemie GmbH, Taufkirchen, Germany), which was transported into the MixM using a syringe pump (Nemesys, Cetoni GmbH, Korbußen, Germany). Starting with the initial resorufin concentration of 100 µg mL^−1^ (TD flow rate of 100 µL min^−1^, MEM flow rate of 0 µL min^−**1**^), the ratio between the TD flow rate and the MEM flow rate was systematically changed. Increments of 10 µL min^−1^ were used, leading to flow rates of 30 µL min^−1^ for TD and 70 µL min^−1^ for MEM. After mixing the resorufin solution (GM, inlet 2) with the MEM (GM, inlets 1, 3), droplet generation was initiated by pumping PFD into inlets 4 and 5 of the GM (see Figure 1D) in a horizontal manner. For each flow rate ratio, 30 droplets were generated using a PFD flow rate of 500 µL min^−1^, resulting in an average droplet volume of 768 nL. The droplets were stored on an SM directly connected to the AM. The SM (Figure 1A) consists of a holder onto which a PTFE tube is spirally wound. Through the PTFE tube, the droplets were directed to the AM for subsequent spectroscopic resorufin detection, as described above.

### 2.8. Calibration of Active Fluid Injection Using the CM

To calibrate the active fluid injection, droplets were generated using the GM with flow rates of 150 µL min^−1^ for MEM and 250 µL min^−1^ for PFD. This resulted in droplets with an average volume of 787 nL. The distances between the droplets were increased by introducing an additional DM (see Figure 1B), as detailed in [27], where one side channel was closed. By pumping PFD into the other side channel at a flow rate of 300 µL min^−1^, the distances between the droplets in the sequence were increased. The droplets were then transported into an SM, and droplet lengths were measured using the MicM by guiding them through the ROI of the camera at a flow rate of 50 µL min^−1^. Following this first measurement of droplet lengths using the software MedeaLab (version 1.0) of Medea AV Multimedia and Software GmbH, Nürnberg, Germany (see Section 2.5), the resorufin was injected into the droplets using the CM.

For the calibration of the CM, both the opening time and the pressure difference were varied. Initially, the opening time of the piezo valve was varied between 1 ms and 8 ms in 1 ms steps at a constant pressure difference of 800 mbar. Afterwards, the pressure difference between the reservoir and the microchannel was varied between 400 mbar and 1800 mbar in 200 mbar steps with a constant opening time of 5 ms. The fluorescence signal of each droplet was measured, as described above, at 586 nm. An immersion fluid was not used in these experiments. Subsequently, the droplet lengths were once again determined (version 1.0) and compared to the first measurements. The difference in droplet volumes, representing the injected volume, was calculated and subsequently compared to the measured fluorescence intensity. 

### 2.9. Generating a Cell Gradient in a Droplet Sequence Using the GM

To demonstrate the capability of the GM to generate droplets with different defined cell concentrations, a cell suspension (6.6 × 10^4^ cells mL^−1^) was manually introduced into a MixM. This guarantees the homogenous mixing of cells, a prerequisite for consistent transport to the GM. The MixM consists of a cavity in which the cell suspension is pipetted, and a stirring blade with a constant stirring velocity of 300 rpm ensures uniform mixing of the cell suspension. The transport of the cell suspension was achieved by displacing the cell suspension with TD (172456, Sigma Aldrich Chemie GmbH, Taufkirchen, Germany), which was propelled into the MixM using the syringe pump (Nemesys, Cetoni GmbH, Korbußen, Germany). Subsequently, the cell concentration in the droplets was adjusted by varying the flow rates of TD (inlet 2; see Figure 1D) as well as the flow rates of MEM using the GM (inlets 1 and 3; see Figure 1D). Starting with a concentration of 6.6 × 10^4^ cells ml^−1^ and flow rates of 100 µL min^−1^ for TD and 0 µL min^−1^ for MEM, the flow rates of TD and MEM were systemically changed. Steps of 20 µL min^−1^ were implemented, concluding on flow rates of 20 µL min^−1^ for TD and 80 µL min^−1^ for MEM cell culture medium. After mixing the cell suspension with the cell culture medium, the droplet generation was initiated by pumping PFD into inlets 4 and 5 (see Figure 1D) of the GM. Using a PFD flow rate of 500 µL min^−1^, 30 droplets were generated for each flow rate ratio, resulting in droplets with an average volume of 768 nL. All droplets were transported into an SM, which was reversibly connected with the GM. For subsequent determination of cell number per droplet, the SM was transferred from the GM to the MicM. For this, the droplets were transported to the visual field of the microscope (BX60, Olympus Deutschland GmbH, Hamburg, Germany) using a manually controlled syringe pump. The cell number per droplet was determined by manually moving the microscopic focus plane along the z-axis through the droplet while counting the cells. The high quality of the optical imaging using the MicM (see Figure 1G) guaranteed the identification of single cells inside the droplets without the need to increase the optical magnification.

## 3. Results 

### 3.1. Characterization of Droplets Generated with the TFP

The first step in droplet-based microfluidic applications is the generation of droplets, which was executed using the TFP. Following droplet generation, the SM was transferred from the TFP to the MicM, to assess droplet lengths as well as the distances between them, as described in Section 2.5. As exemplarily shown in Figure 3, both the droplet lengths and the distance between the droplets could be controlled by varying the flow rates of the TFP. However, it is noteworthy that both parameters could not be independently set. At a constant $\dot{V}$2 flow rate of −89.4 µL min^−1^, a $\dot{V}$1 flow rate of 80 µL min^−1^ resulted in a mean droplet length of 1.27 mm (SD = 0.04 mm), corresponding to a droplet volume of 735 nL. For a $\dot{V}$1 flow rate of 33 µL min^−1^, the mean droplet length was 2.38 mm (SD = 0.05 mm), representing a droplet volume of 1083 nL. Consequently, droplet volumes ranging from 735 nL to 1083 nL can be generated by using the TFP. The lower the $\dot{V}$1 flow rate of the continuous phase at a constant $\dot{V}$2 flow rate, the higher the droplet volume.

### 3.2. Influence of Water as Immersion Fluid for Fluorescence Detection with the AM

**Figure 4:**
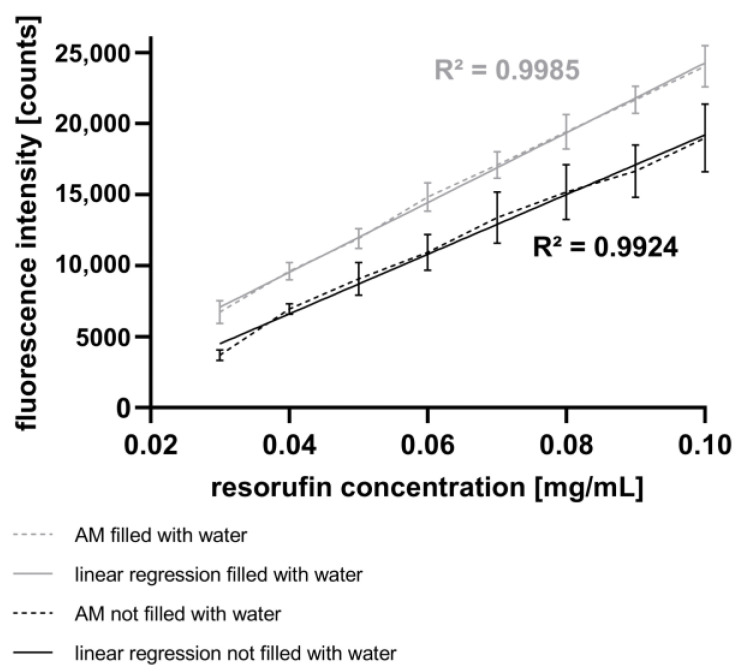
Fluorescence intensity of the droplets measured with the AM shown as mean with SD, n = 90. Resorufin concentrations were varied using the GM. Measurements without water as immersion fluid in the AM (black) are compared to the measurements with water as immersion fluid (grey).

In this study, spectroscopic measurements of fluorescence intensity were performed (see Section 2.7). Figure 4 presents the fluorescence intensities corresponding to different resorufin concentrations in droplets generated using the GM. The high linearity and low standard deviation attest to the high quality of the GM and the AM. Additionally, Figure 4 illustrates the impact of water as an immersion fluid in the AM. Compared to the experiments without water as an immersion fluid, the fluorescence intensities increase, and the signal deviations decrease in experiments performed with water as the immersion fluid. Likewise, the signal deviation rises with increasing fluorescence intensity, suggesting a reduction in signal reproducibility.

### 3.3. Accuracy of Injected Volumes by Fluid Injection Using the CM

To add different but defined volumes into an existing droplet sequence, a reproducible merging, here through active fluid injection, is required. To analyze the viability of cells during drug testing, e.g., resazurin-based alamarBlue™, it is necessary to inject defined volumes of drugs as well as assay reagents into the droplets. Merging of droplets offers several advantages in terms of droplet handling compared to conventional mixing methods [13]. One example of active merging shows that by heating the micro-channel with a laser beam, one droplet stopped, as the result of the Marangoni effect, followed by merging with the subsequent droplet [42]. Without the use of surfactants, this strategy could also lead to unintended merging of several droplets.

To overcome unintended droplet merging, we implemented active fluid injection with the CM using a piezo valve, as described in Section 2.8. 

**Figure 5:**
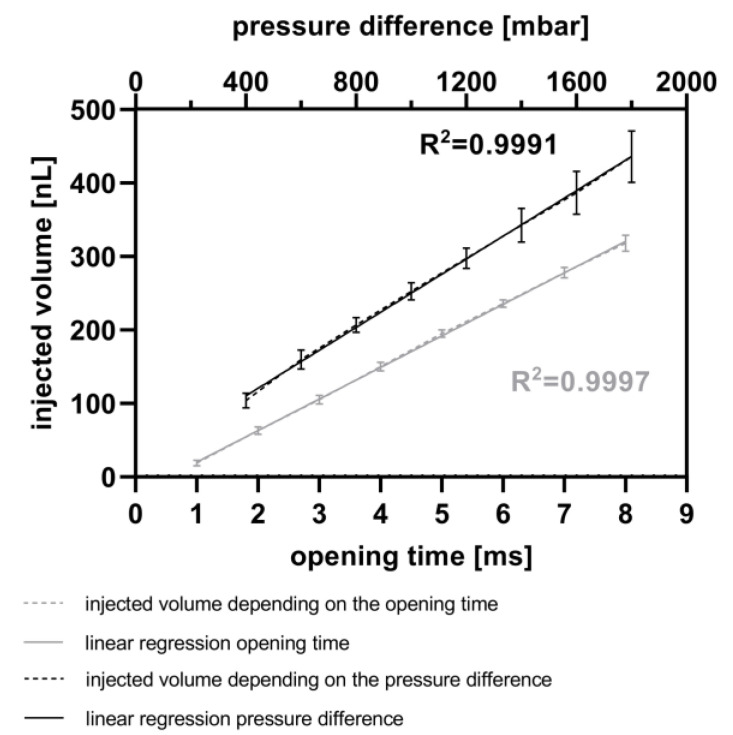
Calibration of injected volume. Variation of the opening time from the piezo valve (ranging from 1 to 8 ms) at a constant pressure difference of 800 mbar between the reservoir and the micro-channel (grey). Variation of the pressure difference between 400 and 1800 mbar at a constant opening time of 5 ms (black). Each calibration point comprises up to 90 droplets, and comparisons were made before and after injection of resorufin fluorescence dye.

Figure 5 illustrates the calculated injected volume of resorufin solution, based on measurements of droplet length, using the MedeaLab software (version 1.0) by Medea AV Multimedia and Software GmbH. It shows the ability to inject fluids into droplets with a very high linearity (R^2^ = 0.9991) by varying the pressure difference between the reservoir and the micro-channel. This difference was varied from 400 mbar to 1800 mbar, with a constant piezo valve opening time of 5 ms. Under these parameters, it is feasible to inject between 103 nL (400 mbar, 5 ms) and 435 nL (1800 mbar, 5 ms) of resorufin into the droplets. Each data point in the graph represents three experiments, each with 30 droplets per injection parameter. The standard deviation ranges from 10 nL to 14 nL between 400 mbar and 1200 mbar pressure differences and from 23 nL to 35 nL between 1400 mbar and 1800 mbar pressure differences. Higher pressure differences resulted in droplet movement due to the pulses, potentially leading to decreased accuracy of the injected volume. Furthermore, Figure 5 demonstrates a high linearity (R^2^ = 0.9997) for fluid injection into droplets at piezo valve opening times ranging from 1 ms up to 8 ms, with a constant pressure difference of 800 mbar between the reservoir and the micro-channel. With these parameters, fluid volumes ranging from 19 nL (1 ms) up to 318 nL (8 ms) were injected into the droplets. The very low standard deviations range from 4 nL (1 ms piezo valve opening time) to 11 nL (8 ms piezo valve opening time).

### 3.4. Comparison of the Fluorescence Intensity and the Resorufin Concentration of Droplets after Active Fluid Injection with the CM

**Figure 6:**
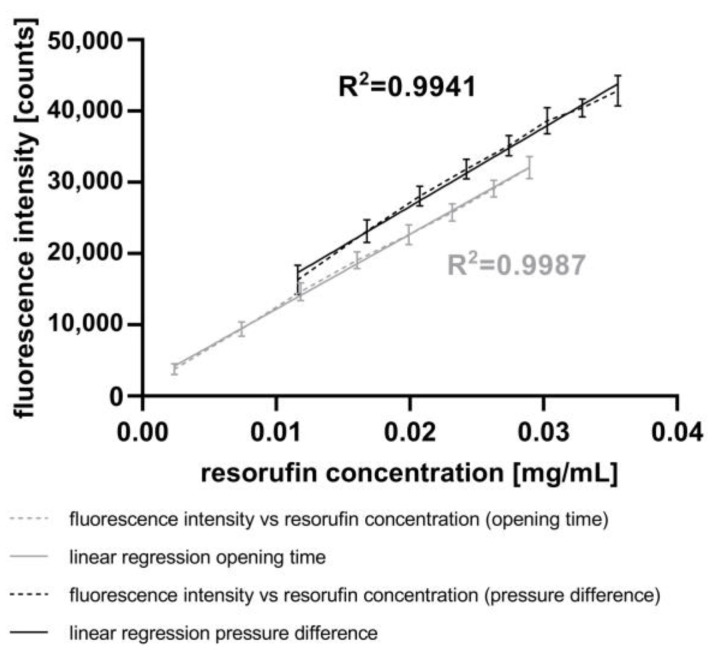
Correlation between the injected resorufin concentration and the fluorescence intensity measured using the AM. The injected resorufin volume was controlled through either variation of the pressure difference (black) or variation of the opening time of the piezo valve (grey) both manipulated using the CM.

To validate the measurement of the increasing volume of the droplets (see Section 2.5), we measured the fluorescence intensity in droplets after the injection of resorufin. Figure 6 depicts the correlation between the averaged fluorescence intensity and averaged resorufin concentration inside the droplets using either varying pressure differences or piezo valve opening times, as described above. The resorufin concentrations were calculated based on (i) the averaged droplet volume before the resorufin injection, (ii) the averaged injected volume, and (iii) the concentration of the stock solution. 

Applying the lowest pressure difference of 400 mbar at a constant piezo valve opening time of 5 ms, the fluorescence intensity of 16,323 counts corresponds to a resorufin concentration of 0.0116 mg mL^−1^. The highest pressure difference of 1800 mbar resulted in a fluorescence intensity of 42,850 counts, corresponding to a resorufin concentration of 0.0356 mg mL^−1^. The coefficient of linearity (R^2^) of 0.9941 shows a high correlation between both parameters.

The results obtained by varying the opening times of the piezo valve at a constant pressure difference of 800 mbar show the same trend. With the lowest opening time of 1 ms, the fluorescence intensity was 3766 counts, corresponding to a resorufin concentration of 0.0024 mg mL^−1^. In contrast, the highest opening time of 8 ms resulted in a fluorescence intensity of 32,043 counts, corresponding to a resorufin concentration of 0.0289 mg mL^−1^. The correlation between both these parameters is highly linear, with a coefficient of linearity (R^2^) of 0.9987. Compared to the experiments with varying pressure differences, the experiments with varying piezo valve opening times achieved smaller variances in standard deviations.

Therefore, to achieve a variation in the fluorescence intensity of the droplets through the adjustment of injection volumes, it is recommended to vary the piezo valve opening time rather than the pressure differences. Furthermore, the results from the experiments show that the piezo valve operates with highest accuracy at lower pressure differences.

### 3.5. Characterization of a Cell Concentration Gradient of U87MG ATCC Cells in Droplets

**Figure 7:**
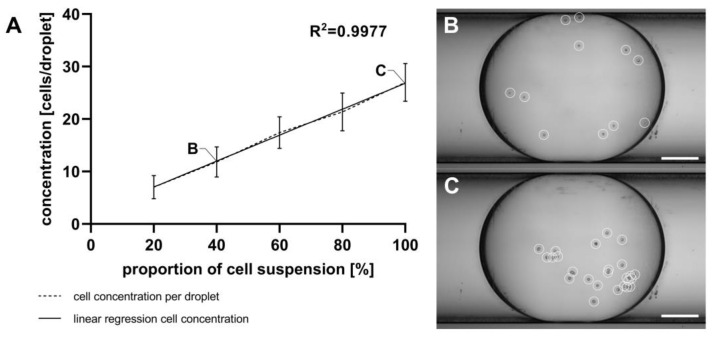
(**A**) Quantification of U87MG ATCC cells within droplets generated using the GM and manually counted using the MicM, shown as mean with SD, n = 30. Different cell concentrations were achieved by adjusting the flow rates of the cell suspension and cell-free medium. (**B**) Extended depth of field (EDF)-projection from an exemplarily shown droplet containing 11 U87MG ATCC cells, (**C**) EDF-projection from another exemplarily shown droplet containing 25 U87MG ATCC cells; cells are highlighted with white circles. Scale bar = 250 µm.

For reliable results in cell viability measurements using resazurin-based dyes like alamarBlue™, it is necessary to calibrate the linear range of the assay. This calibration involves creating a linear cell gradient within a droplet sequence. Droplet generation from the cell suspension using the GM and cell counting with the MicM were performed as described in Section 2.9. Figure 7 shows the attained cell concentrations per droplet, determined through manually counting with a microscope. While there is a high level of linearity across the five cell concentrations, a notable inconsistency between the theoretical cell numbers (calculated based on the concentration of the initial cell suspension) and the actual cell numbers was found. Depending on the flow rate ratio, there should be 10 to 50 cells per droplet, which is a very low number of cells. However, the actual count ranged from 7 to 27 cells per droplet.

## 4. Discussion

The first step in droplet-based microfluidic applications is the generation of droplets, and this plays a pivotal role, making the choice between passive and active methods crucial [43]. While a majority of techniques passively generate a continuous stream of evenly spaced droplets with volumes featuring standard deviations ranging from less than 2% [43] to 2.5% or 5% [27], this study utilizes the TFP for droplet generation. The TFP, by allowing controlled shaking during the droplet formation process, ensures reproducible cell concentrations over time. This eliminates the need for specially developed stirrers, which are required in combination with microfluidic chips to maintain reproducible cell concentrations throughout an experiment [44]. 

The ability to vary cell concentrations easily is a key advantage of the TFP. By changing the vessel of the TFP, having different starting cell concentrations becomes a straightforward process. In contrast, in chip-based modules with geometries like T-junctions or flow-focusing designs, flow rates influence cell concentrations [45]. The use of PTFE tubes with an inner diameter of 1 mm as the droplet guiding channel, as used here, allows the cultivation of about 50 to 10,000 cells as initial cell concentrations in the droplets. Maintaining a balanced cell-to-droplet volume ratio is fundamental to ensure a sufficient nutrient supply for cell cultivation, especially in the case of long-term cultivation experiments over several days [46]. The TFP offers flexibility with tubes of varying diameters, increasing the range of possible droplet diameters and volumes that can be achieved [28]. The TFP is faster to produce and easily adaptable, as no new constructions are needed for droplet volume adaptation, as described in flow-focusing solutions using chips [47,48]. The TFP-based droplet generation, as depicted in Figure 3, aligns with numerical studies on chip-based droplet generators, where the droplet volume increases with decreasing flow rate ratios from the continuous to the disperse phase ($\dot{V}$_oil_/$\dot{V}$_water_) [49]. Experimental setups using a T-junction have also found similar results [50]. With increasing droplet volumes (ranging from 735 nL (SD = 3.54%) to 1083 nL (SD = 2.17%)), the distance between the droplets decreases, accompanied with lower standard deviations (SD = 3.44% for droplet distance) when using the TFP. In summary, the TFP provides an easy and efficient way to generate droplets. It requires less preparation compared to chip-based modules, as the components only need to be autoclaved, eliminating the need for plasma functionalization. The standard deviations of droplet volumes, obtained with the TFP, are comparable to those obtained using the DM, while no significant changes in droplet distance and their standard deviations by changing flow rate ratios were found [27,34,35]. 

Mixing methods during droplet generation can be categorized into two main approaches: mixing during droplet generation and mixing of pre-existing droplets, e.g., using meander structures [51]. The generation of droplets with varying cell concentrations can be facilitated using the GM, which mixes a cell suspension with cell culture medium. However, challenges, such as deviations from expected cell concentrations, as described in Section 3.5, can arise due to cell sedimentation and cell adhesion in the PTFE tube [52]. In the presented system, where the cell suspension was guided through the PTFE tube connected to inlet 2 of the GM in a continuous flow, similar problems exist. These effects are caused by the parabolic streaming profile of the cell suspension. Rearranging the GM and the PTFE tube vertically along with reducing the length of the PTFE tube could mitigate these effects. The presented “*pbb*” platform offers the potential to create 3D cell cultures from adherent cell lines to better represent the in vivo situation for personalized medicine compared to commonly used monolayers. Over the years, many geometries have been developed to generate concentration gradients in droplet-based microfluidics [53]. Pneumatic valve-controlled, tree-shaped, and Y-shaped systems have been shown. Pneumatic valve-controlled systems have limitations on the number of channels that can be used to mix fluids. Y-shaped systems do not allow for the determination of defined concentrations. In contrast, with tree-shaped approaches, it was possible to generate droplets of defined concentrations in different channels.

In order to successfully transfer cell-based assays from conventional microwell plates to the “*pbb*” platform, it is essential to analyze the content of generated droplets. One aspect is the measurement of fluorescence intensities corresponding to the amount of fluorescent cells or the concentration of fluorescent products metabolized by cells in droplets, as in assays like in the alamarBlue™ assay [54]. In this study, we present a spectrometer-based AM that is well-suited for fluorescence measurements, exhibiting a linear range at concentrations ranging from 0.03 mg mL^−1^ to 0.1 mg mL^−1^ (see Figure 4). 

Previous studies present different analytical approaches for droplet detection. Contact-free high-throughput detection of droplets has been realized through microscopic techniques [28] or by using a spectrometer [27]. Additionally, calorimetric approaches using a microchip calorimeter have been described [55,56]. Cahill et al. and Cao et al. introduced a specialized flow sensor to measure droplet conductivity, which was developed by S. Wiedemeier [57,58]. Dittrich et al. presented a microfluidic system wherein the fluorescence of GFP was excited using a laser beam, demonstrating a lower limit of 2 nM GFP in aqueous droplets. They found a highly linear relationship between fluorescence intensity and GFP concentrations within the range of 2 nM to 70 nM [59]. 

In addition to analyzing droplets contents, the precise injection of fluid volumes is essential for applications like viability assays, long-term cultivation, or high throughput drug screenings. Different strategies for merging existing droplet sequences have been developed in recent years, encompassing active and passive approaches [60,61,62]. One example is a strategy based on electrical fields. Kielpinski et al. presented a microfluidic device featuring variable merging strategies with an applied electrical field, demonstrating the capability to merge droplets, even those stabilized with surfactants [62]. Another approach involves the use of magnetic particles and the application of a magnetic field to the microfluidic channel [63]. Varma et al. present a microfluidic chip for merging a fluid, containing magnetic particles (ferrofluid) stained with two different dyes to enhance visualization. By adjusting the strength of the magnetic field, it became possible to merge two or more droplets of the ferrofluid [63].

In conclusion, the results obtained using the CM demonstrate the capability to inject fluids into droplets by varying both the piezo valve opening time and the pressure difference in a linear and reproducible manner. Furthermore, the experiments have verified that the AM is well-suited for measuring the fluorescence intensity of droplets resulting from the injection of resorufin dye. A comparison of these results with those using the mixing strategy with the GM (Figure 4) indicates a higher accuracy of the fluorescence intensity with active fluid injection (Figure 6). However, a broader range of chemical concentrations in the droplets was determined with the mixing strategy. This method allows the injection of fluids such as drugs, dyes, and assay reagents into droplets, similar to the process with pipettes in microplates. In contrast to passive merging strategies, active merging needs an external energy for the fusion of droplets.

The data presented in Figure 5 introduce another way to actively inject fluids into droplets using a piezo valve without the need for stabilizing the droplets with surfactants. Other workgroups use piezo valves for droplet generation with a linear relationship between the opening time of the piezo actor and the droplet volume [64]. With this chip, they are able to generate droplets with comparably higher volumes (200–1800 nL). In contrast, using the CM, we are able to inject small volumes ranging from 19 nL to 435 nL in a linear fashion and with low standard deviations (4 nL to 35 nL). 

In contrast to mixing strategies using specific micro-channel geometries [60,65,66], the CM enables initial complete mixing after fluid injection without need for special geometries. This feature overcomes problems like pressure differences in passive merging, as reported by Gunda et al. [67]. In the CM, the position of the actor of the piezo valve can be adjusted with a control unit. Subsequently, the actor of the piezo valve has the same displacement, leading to more reproducible results at higher pressures. The applied pressure in the reservoir is directly controlled by the measured system pressure; hence, there are no problems with leakages during the fluid injection in the “*pbb”* system, as described from Bußmann et al. for some of their valve arrangements [68]. Another notable advantage of the CM module is its injection rate, allowing droplet merging at flow rates of 300 µL min^−^^1^. This is crucial for performing cell viability assays, as the measurement is subjected to strict time constraints. With these parameters, the platform can merge about 600 droplets in 8 min. The GM further complements the system by enabling the generation of droplets with different contents and minimizing dead volume due to the direct connection of the mixing area to the droplet generation area. This is an advantage of modular microfluidics compared to monolithic systems [29]. While mixing and passive merging have the advantage that no additional equipment is needed, there are several drawbacks, like pressure variations in the system, that could inhibit the merging, resulting in incomplete fusion of fluids or unintended droplet merging. The use of surfactants in these approaches also imposes limitations on merging droplets without external energies [69].

With the demonstrated high accuracy of the modules, all process steps to implement resazurin-based viability assays using the *“pbb”* platform could be prepared. Because the droplets are serially arranged in the tubes, each droplet has its own identity. Therefore, in each droplet, one specific process can be investigated and changes can be observed over time. This is made more complex using surfactants or when a high number of droplets is collected in one chamber with special geometries [70,71]. In recent years, the modular droplet-based microfluidic platform was upgraded to incorporate new process steps. The GM now enables the generation of droplets from three different mixed watery phases within a single microfluidic module. The CM was developed to actively inject fluids into an existing droplet sequence in flow, leading to initial complete mixing of different reagents. Consequently, mixing strategies inside droplets are negligible, aligning with findings in numerical studies described by Mbanjwa et al. and Rezaeian et al. [72,73]. The CM essentially serves as the pipette in the modular droplet-based system. The AM is designed for measurements of fluorescence intensity and absorbance in droplets. Additionally, with the MicM, the refractive indices of the tubing and their environment were adjusted to optimize microscopic imaging in droplet-based microfluidics.

## 5. Conclusions

High-precision microfluidic modules, developed specifically for droplet-based microfluidics, have been successfully manufactured using polymers and were surface-treated by plasma deposition procedures. These modules have been integrated as functional components of the “*pbb*” platform to realize droplet-based microfluidics for applications using cells suspensions. To optimize the microfluidic modules, experiments for droplet generation, droplet conditioning, and droplet characterization were performed. The study demonstrated that it is possible to generate droplets consisting of human cell cultures and to establish cell gradients within droplets using the GM. Additionally, the MicM demonstrated the ease with which the droplet-based microfluidic platform can be connected to peripheral devices such as microscopes. The CM provides a versatile way to inject various fluids into droplets (e.g., drugs, dyes) in a reproducible and linear manner. Pressure irregularities in the fluid system are negligible, as the pressure difference between the droplet-containing micro-channel and the reservoir, which is connected to the piezo valve, is measured and regulated. Together with the development of new modules for droplet generation (GM), active fluid injection (CM) droplet microscopy (MicM), and spectroscopy (AM), it will be possible to realize the transfer of cell-based assays from microwell plates to the “*pbb*” system in the near future. The modular nature of the microfluidic platform opens up an enormous potential for a wide range of applications. As the “*pbb*” platform is a closed system, it minimizes the risk of contamination, making the platform suitable for applications and manipulations both outside clean benches and with hazardous germs.

## Data Availability

The data presented in this study are available on request from the corresponding author. The data are not publicly available due to privacy restrictions.
